# A snapshot of Iraqi psychiatry

**DOI:** 10.1192/bji.2020.19

**Published:** 2021-02

**Authors:** Aws Sadik

**Affiliations:** Core Psychiatry Trainee, Avon and Wiltshire Mental Health Partnership NHS Trust, Bath, UK. Email: a.sadik@nhs.net

**Keywords:** Transcultural psychiatry, low and middle income countries, education and training

## Abstract

During a visit to Baghdad, I was able to visit several key centres in Iraqi psychiatry: the Ministry of Health, Baghdad General Hospital, Ibn Rushd Hospital and Al Rashad Hospital. This was my first experience of mental healthcare outside England, and it left me with a range of discussions and experiences to reflect on. I hope that this article offers a fair flavour of Iraqi psychiatry from the perspective of a UK-trained doctor.

‘I feel like I am not really alive in this country. What kind of country is this? Endless violence, no jobs, no future. It makes me depressed.’

In Al-Rashad Hospital's busy out-patient clinic, it was difficult to determine whether this 20-year-old gentleman was experiencing depersonalisation or simply using powerful language in describing the state of the nation.

The turmoil of Iraq's recent history is well documented.^1^ At the time of my arrival, the potential fallout from Qasem Soleimani's assassination at Baghdad International Airport was awaited nervously. However, this was a country already on edge: our 20-year-old gentleman was wearing a forearm cast after being injured at Baghdad's Tahrir Square, the epicentre of months of nationwide protests against corruption and unemployment. Protests that had been responded to by the killing of unarmed protesters and the promised resignation of the Prime Minister.

The primary purpose of my brief trip to Baghdad was catching up with family; however, it also offered the opportunity to explore transcultural psychiatry. Thanks to the warm and welcoming National Advisor of Mental Health, Dr Emad Abdulrazaq, I was able to visit the psychiatric in-patient ward at Baghdad General Hospital, as well as Iraq's only two mental health hospitals: the short-stay Ibn Rushd Hospital (50+ beds) and the longer-stay Al-Rashad Hospital (1200+ beds).

Over glasses of sweet tea, the leaders of these units generously offered me insights into psychiatry in Iraq compared with the UK. Richness was then added to these insights through meeting out-patients at Al-Rashad Hospital. Pertinent, mostly paraphrased, quotes from those I met will provide the framework for this reflection. Details of the patients discussed in this essay have been fully anonymised.

‘*Please admit my daughter*.’

Although core principles are similar, service models differ considerably between the two nations. In the UK, mental healthcare is mostly carried out by general practitioners and community teams, both of which are essentially non-existent in Iraq. Instead, psychiatric services are limited to 34 out-patient clinics, 21 in-patient wards within general hospitals, and two Baghdad mental health hospitals. Iraq has approximately 100 psychiatrists, so 0.34 per 100 000 population^2^ in comparison with 8 per 100 000 in the UK. This disparity is of a higher ratio than that seen with physicians: 82 per 100 000 *v.* 280 per 100 000, respectively.^3^ Factors keeping numbers low include stigma and a relative lack of the private practice opportunities on which many of Iraq's doctors rely.

Family is central to mental healthcare in Iraq. Unwell relatives tend to be cared for at home, without community health or social care support. In hospitals, in-patients often have relatives staying overnight and assisting with nursing care. Discharge tends to rely on the availability of a family home. Those without family support are present in larger proportions at the longer-stay Al Rashad Hospital, where many will reside until the end of their lives, as homelessness is the only available discharge destination.

Despite the quoted father's desire to see admission, this would need to occur voluntarily or via a court. The Iraq Mental Health Act 2005 is heavily influenced by British Mental Health Acts, with similar provisions for compulsory admission by psychiatrists.^4^ However, these powers are not used, for reasons including a lack of historical precedent and security risks to clinicians. Instead, courts determine involuntary admission with the support of expert psychiatric committees.

‘*She was married at fourteen years old. … The in-laws have banished her from their home, and she has not been allowed to see her child for seven months now.*’

Observance of the rights of women and girls is limited by international standards, and there is a risk of further backwards steps.^5^ Traumas such as the separation of a mother and baby can add to the psychological burden of wars and insecurity, and had possibly contributed to the seizures that had brought this young mother to the clinic.

Stigma around mental illness was a major theme of my discussions and potentially also a factor in this teenager's heart-breaking story. ‘Faith healers’ are often sought before a psychiatrist^6^ and erroneous perceptions are common, for example, ‘sedatives are all that psychiatrists can offer’.

‘*He first took escitalopram but the pharmacy stock ran out, so we switched to amitriptyline. That is not currently available, so we'll prescribe fluoxetine.*’

Commonly used psychotropic medications are included in Iraq's official lists of essential and important medications that should be available at public hospital pharmacies. However, particularly at Al-Rashad Hospital, where the prescription load is high and locals cannot afford to pay for medications elsewhere, switching medications may be mandated by stock rather than clinical considerations. As well as the likely detrimental effect on medication concordance, variable supplies can make national clinical guidelines more difficult to design and adhere to.

‘*I recommend getting the branded olanzapine instead; this one was made in Mosul.*’

Substandard and falsified medications are a global concern. In contrast to the relatively strong regulation of medications in the European Union, medications in low- and middle-income countries are more likely to vary in composition and effectiveness.^7^ ‘Branded’ medication supplies fluctuate even in private pharmacies and so individuals may rely on substandard alternatives, risking a deterioration in their mental health.

‘*There is a well-designed de-escalation room, but rheumatology has that part of the hospital now.*’

Bed pressures represent a shared theme between our nations. Mental health facilities may have to be squeezed in order to accommodate other stretched specialties, with unfortunate outcomes including shared patient bays and the loss of de-escalation rooms. That said, the hospitals do also have services that would not always be seen in UK units: in-house pharmacy and pathology laboratories, and electroencephalogram, therapies, and electroconvulsive therapy suites. I was particularly impressed that Ibn Rushd Hospital has a shared IT system for clinical documentation and investigation results – no need for the ‘copy-and-paste’ that is a key competency for many British trainees.

‘*I can't remember what the Turkish psychiatrist said.*’

Health tourism is a major activity among Iraqis, with many travelling across the Middle East and Asia in order to access treatments either unavailable in Iraq or perceived to be higher in quality elsewhere. Potential weaknesses of this approach include language barriers, limited follow-up and vulnerability to profit-driven overtreatment. In the quoted case, a father had sought the opinions of psychiatrists across the Middle East, leading to a questionable diagnosis of childhood schizophrenia before puberty and years of antipsychotic use – not necessarily from a Turkish psychiatrist, I hasten to add.

‘*I didn't take anything, just smoked a tobacco waterpipe with my friends.*’

Substance misuse has historically been perceived to be relatively low in Iraq, particularly compared with neighbouring Iran. However, poverty and more open borders have contributed to rising drug use in recent years.^8^ Crystal methamphetamine use, in particular, has been garnering great attention and concern.^9^ It was highlighted to the quoted teenager that anything may be unknowingly be inserted into a waterpipe. However, later in the day, a message arrived to say he had admitted to having taken five pink pills the day before.

‘*Please look at this X-ray, Doctor.*’

Many UK psychiatrists would be bemused at a request to report a physical copy of a pelvis X-ray. Unfamiliarity with radiology reporting is probably linked to ease of access to a radiologist and the inability to access images located on separate IT networks. In Iraq, images tend to be carried by patients in physical form, and review by a psychiatrist may be the most practical option. Interestingly, on this occasion at least, it was the most senior psychiatrist that was sought, rather than the juniors, who would have most recently had experience of the relevant specialties.

‘*Recent ministers have shown great support for this hospital.*’

It was reassuring to hear positivity regarding mental health policy. Iraqi mental healthcare continues to evolve. An update to the Mental Health Act should soon be ratified. More equitable and efficient primary care models are being developed,^10^ and rehabilitation units and residential homes are planned. Subspecialty training programmes are being strengthened, and curricula are being formalised for mental health nurses, psychologists and social workers. Importantly, sustained advances will require national security and effective investment in public services, rather than the stifling sectarianism and corruption that have defined Iraq this century.

‘*How can we help you?*’

I started my visits with the vague purpose of seeing what psychiatry was like in Iraq and feel that I have benefited from the experience in many ways. The ability of psychiatry to evolve for the better relies on questioning our practice, engaging criticism and understanding our society. Peering at a different system has allowed me to assess more freely the dogmas and values of my work and to consider why particular practices have developed. I feel that I have a better sensitivity to diversity and cultural factors and enhanced motivation for strengthening language skills and learning about other mental healthcare systems. Now that I have had my introduction to Iraqi psychiatry, I hope that cross-border collaboration can be a feature of my career, as psychiatry is particularly exciting when the context changes and experiences are shared with those from other backgrounds.

## Supplementary material

For supplementary material accompanying this paper visit https://doi.org/10.1192/bji.2020.19. click here to view supplementary material
