# A snapshot of Iraqi psychiatry – CORRIGENDUM

**DOI:** 10.1192/bji.2020.25

**Published:** 2022-08

**Authors:** Aws Sadik

**Keywords:** Transcultural psychiatry, low and middle income countries, education and training

This article understates the number of psychiatrists and psychiatric hospitals in Iraq, appearing to exclude figures from the Kurdistan Region. In addition to the figures stated in the article, there are at least 40 qualified psychiatrists, three specialised mental health hospitals (for 40–60 patients) and two other psychiatric units inside general hospitals within Kurdistan. The number of consultant psychiatrists across Iraq appears to be over 160. I am grateful to Prof. Nazar M Mohammed Amin (University of Sulaimani) for providing this information.
